# Supplementary figures and images for: Wolbachia strain wAu efficiently blocks arbovirus transmission in Aedes albopictus
Source: PLoS Negl Trop Dis. 2020 Mar 10;14(3):e0007926. doi: 10.1371/journal.pntd.0007926 (PMC7083328; doi:10.1371/journal.pntd.0007926)

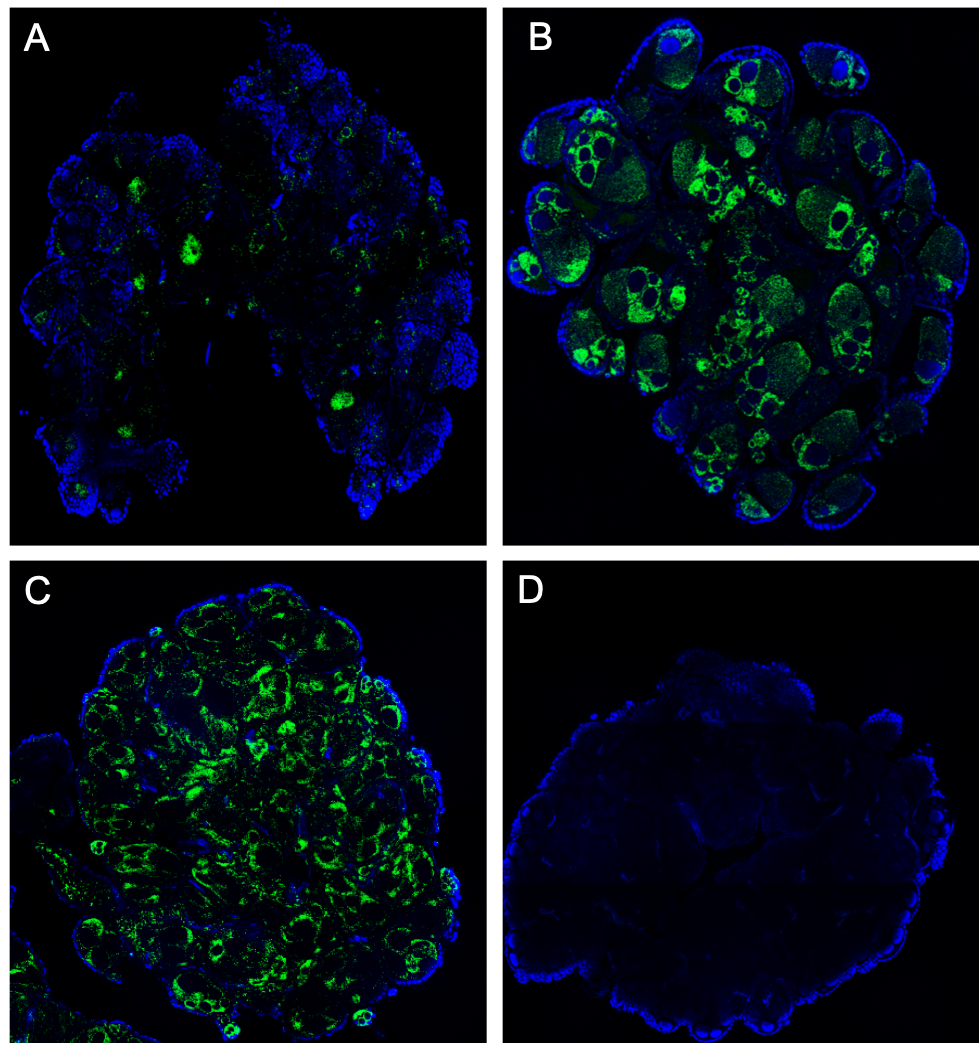

Supplement: S1 Fig — Visualization of distributions of general Wolbachia (green) in ovaries of 5-day old females from the wild-type (A), JF.wAu (B), Ae. aegypti-wAu lines (C) and a no-probe control on wild-type (D). Blue stain is DAPI. (TIF) [file pntd.0007926.s001.tif]
