# Supplementary material for: Wolbachia strain wAu efficiently blocks arbovirus transmission in Aedes albopictus
Source: PLoS Negl Trop Dis. 2020 Mar 10;14(3):e0007926. doi: 10.1371/journal.pntd.0007926 (PMC7083328; doi:10.1371/journal.pntd.0007926)
Supplement: S1 Table — (DOCX) [file pntd.0007926.s002.docx]

Table S1: List of sequences of oligonucleotides and probes**.**

| **Primer name** | **5’-3’ Sequence** |
| --- | --- |
| *w*AlbA-F [17] | GTAGTATTTACCCCAGCAG |
| *w*AlbA-R [17] | ATCTGCACCAGTAGTTTCG |
| *w*AlbB-F [17] | GCAATACCTATGCCGTTTA |
| *w*AlbB-R [17] | GACGAAGGGGATAGGTTAATATC |
| Au F (18F) [17] | TGGTCCAATAAGTGATGAAGAAAC |
| AuWSP R [17] | TTTGCTGGGTCAAATGTTACATCTT |
| qHTH-F [50] | TGGTCCTATATTGGCGAGCTA |
| qHTH-R [50] | TCGTTTTTGCAAGAAGGTCA |
| qWSP-F [16] | ATCTTTTATAGCTGGTGGTGGT |
| qWSP-R [50] | GGAGTGATAGGCATATCTTCAAT |
| qAlbAF | CAAGAATTGGCGGGCATTGA |
| qAlbAR | AATGTTGCACCACCAACGTC |
| 183F [50] | AAGGAACCGAAGTTCATG |
| QBrev2 [51] | AGTTGTGAGTAAAGTCCC |
| qwAuTF | CGAAATTAGTTGGTTTGGTGC |
| qwAuTR | GCTTGTAGTTTATGCTTTCTCTC |
| AlboRpS17-F [52] | GAACGACAGCAGCGAAACTT |
| AlboRpS17-R [52] | GTCACGAAACCAGCGATCTT |
| DENV-NS5-F [16] | ACAAGTCGAACAACCTGGTCCAT |
| DENV-NS5-R [16] | GCCGCACCATTGGTCTTCTC |
| ZIKV-835 [53] | TTGGTCATGATACTGCTGATTGC |
| ZIKV-911c [53] | CCTTCCACAAAGTCCCTATTGC |
| wsp probe W2[16] | 5’-CTTCTGTGAGTACCGTCATTATC-(Alexa Fluor 488)-3’ |
| wsp probe W3 [16] | 5’- AACCGACCCTATCCCTTCGAATA-( Alexa Fluor 488)-3’ |
